# Association Between PON1 (L55M and Q192R) Genetic Polymorphism and Recurrent Pregnancy Loss in North Indian Women Exposed to Pesticides

**DOI:** 10.1055/s-0041-1736342

**Published:** 2021-12-06

**Authors:** Shyam Pyari Jaiswar, Apala Priyadarshini, Apurva Singh, Mohd Kalim Ahmad, Sujata Deo, Pushpa Sankhwar

**Affiliations:** 1 Department of Obstetrics and Gynecology, King George's Medical University, Lucknow, Uttar Pradesh, India; 2 Department of Biochemistry, KGMU, Lucknow, Uttar Pradesh, India

**Keywords:** recurrent pregnancy loss, paraoxonase 1, organophosphate, pesticides, oxidative stress, perda de gravidez recorrente, paraoxonase 1, organofosfato, pesticidas, estresse oxidativo

## Abstract

**Objective** The aim of the present study was to examine the relation between the PON1 polymorphisms and recurrent pregnancy loss (RPL).

**Methods** In a cross-sectional study, blood samples were collected from 100 females. DNA was extracted and PON1 genotypes were determined by polymerase chain reaction (PCR) amplification.

**Results** Regarding PON1 L55M, the mutated allele (M) frequency was found in 70.5% in RPL and in 53.5% in controls; the M allele was signiﬁcantly associated with an increased risk of RPL (adjusted odds ratio [OR _adj]_ = 2.07; 95% confidence interval [CI]; *p* < 0.001). However, regarding PON1 Q192R, the R mutated allele frequency was found in 28.5% in RPL and in 33% in controls. The R allele did not show any risk for RPL (OR _adj_ 0.81; 95%CI; *p* = 0.329).

**Conclusion** The present study suggests that there is an effect of genetic polymorphism on RPL and provides additional evidence that combines with the growing information about the ways in which certain PON1 genotypes can affect the development of the fetus in the uterus.

## Resumo

**Objetivo** O objetivo deste estudo foi examinar a relação entre os polimorfismos PON1 e perda recorrente de gravidez PRG.

**Métodos** Em um estudo transversal, foram coletadas amostras de sangue de 100 mulheres. O DNA foi extraído e os genótipos PON1 foram determinados por amplificação por PCR.

**Resultados** Com relação ao PON1 L55M, a frequência do alelo mutado (M) foi encontrada em 70,5% no PRG e em 53,5% nos controles; o alelo M foi significativamente associado a um risco aumentado de PRG (odds radio ajustado [ORadj] =2,07; intervalo de confiança [IC] 95%; *p* < 0,001). No entanto, em relação ao PON1 Q192R, a frequência do alelo mutado R foi encontrada em 28,5% no PRG e em 33% nos controles. O alelo R não mostrou qualquer risco para PRG (ORadj 0,81; IC 95; *p* = 0,329).

**Conclusão** O presente estudo sugere que há um efeito do polimorfismo genético sobre PRG e fornece evidências adicionais que se combinam com as informações crescentes sobre as maneiras pelas quais certos genótipos PON1 podem afetar o desenvolvimento do feto no útero.

## Introduction

Recurrent pregnancy loss (RPL) is deﬁned as the occurrence of three or more spontaneous miscarriages. It is a diverse condition that involves various etiological factors. Still the precise reason remains ambiguous in more than half of the cases. 1 Between 30 and 50% of conceptions are said to be missed during the 1 ^st^ trimester of pregnancy. Most miscarriages occur around the time of implantation and many times go unnoticed by the pregnant women themselves, who often mistake them for delayed menstruations. Also, between 10 and 12% of all the clinically recognized pregnancies end up as miscarriages during the 1 ^st^ trimester. 2 Despite the fact that chromosomal abnormalities are implicated in ∼ 50% of all miscarriages, the etiology of the other 50% is not exactly identified and may involve anatomic, genetic, endocrine, immunological, and environmental factors. 3 Even after a complete evaluation, 50% of couples remain without a diagnosis for their RPL. Oxidative stress is one of the crucial factors that play a prominent role in the toxicity of various chemical families of pesticides, including organophosphate pesticides. 4 The most largely distributed environmental toxin is pesticides. Even though each one of us is exposed to these xenobiotics to an extent, a few people, like farmers and ﬂoriculturists, are much more exposed to these toxins. These pesticides are extremely noxious to humans. Considerable morbidity and mortality is associated with pesticide poisoning, particularly in developing countries like India, where the pattern of pesticide use is different. 5 The genetic polymorphisms as modifiers of human health diseases have gained attention in recent years, and there is an increased interest in conducting studies to explore the gene-environment interactions to detect susceptible populations prone to develop health problems due to chemical exposure. 6

Amongst the candidate genes which aim to modify vulnerability to pesticides is the one coding for the paraoxonase 1 enzyme (PON1). The PON1 enzyme is an arilesterase class A enzyme that catalyzes the hydrolysis of a wide range of aromatic esters and phosphoesters, which play a multifunctional role in various biochemical pathways such as guarding against oxidative damage and lipid peroxidation, involvement in innate immunity, detoxification of reactive molecules, bioactivation of drugs, modulation of endoplasmic reticulum stress, and regulation of cell proliferation/apoptosis. As they can execute manifold self-governing and often unrelated functions, they are considered “moonlighting proteins.” 7 8

A few findings proposed that several individuals with peculiar genotypes for PON1 have low plasma levels of this enzyme. 9 10 The *PON1* gene comprises a family which incorporates *PON2* and *PON3* , positioned on the long arm of the human chromosome 7(q21.3–22.1). The *PON1* gene contains two polymorphisms in the coding region, one in position Q192R and the other in position L55M. The polymorphism at Q192R gives rise to two alloenzymes which show evident difference in hydrolyzing organophosphate compounds. 11 The activity of *PON1* is substrate dependent; in-vitro analysis revealed that the pace of paraoxon hydrolysis by alloenzyme Q is slower when compared with alloenzyme R, while the rate of diazoxon hydrolysis by alloenzyme R is lower than that of alloenzyme Q. 12 Moreover, the PON1 enzyme assists in the detoxiﬁcation of certain organophosphate pesticides (OP), which are prominent endocrine disruptors and efficiently cross the placental barrier, influencing fetal development. 13 Certain studies have investigated the role of PON1 enzyme activity or genotype and OP pesticide exposure on birth outcomes. 14 15 16 However, the potential association between *PON1* polymorphisms, pesticide exposure, and the risk of miscarriage has not been documented. Hence, the present study aims to evaluate the role of PON1 gene polymorphism in recurrent pregnancy loss.

## Methods

This was a case control study conducted at Queen Mary's Hospital, King George's Medical University, Lucknow, UP, India, from January 2016 to December 2018. A total of 100 women (cases) with a history of at least three recurrent pregnancy losses before the 20 ^th^ week of gestation were included in the present study. An equal number of women (100) undergoing normal vaginal labor at term with live healthy birth were recruited for the control group. All subjects were informed and gave written consent to participate in the study. The present study was conducted in accordance with the guidelines set up by the institutional Ethical committee. (ECR/262/Inst/UP/2013).

Venous blood (2 ml) was drawn from each subject at the time of recruitment and collected in tubes with ethylenediaminetetraacetic acid (EDTA) (1mg/ml). The plasma was immediately separated by centrifugation at 3,500rpm for 15 minutes at 4°C. The cell pack was stored at -70°C until DNA extraction.

Genomic DNA was extracted using the phenol-chloroform method and purified DNA was stored at -20°C until further polymerase chain reaction (PCR) analysis. The *PON1* genotypes were determined by PCR amplification using primers PON1 _192_ (forward) 5′TATTGTTGCTGTGGGACCTGAG3′; PON1 _192_ (reverse) 5′CACGCTAAACCCAAATACATCTC3; PON1 _55_ (forward) 5′CCTGCAATAATATGAAACAACCTG3′; and PON1 _55_ (reverse) 5′ TGAAAGACTTAAACTGCCAGTC3′. The amplification cycle was performed on a thermal cycler under the following conditions: denaturation for 5 minutes at 94°C, followed by 30 cycles of 30 seconds at 94°C, 30 seconds at 61°C, 1 minute at 72°C and, finally, 7 minutes at 72°C. The PCR products were digested with BspPI for P PON1 _192_ and HinIII for PON1 _55_ .

## Results

### Demographic Characterization

There were 100 subjects each in cases and controls group. Both groups were similar regarding age, body mass index (BMI), source of drinking water, and socioeconomic status. There was no significant difference in the mean age between cases and controls ( *p* > 0.05). There was no statistically significant difference in the sociodemographic variables between cases and controls ( Table 1 ).

**Table 1:** Demographical characteristics of women with RPL (case) and control women

Parameters	Cases ( *n* = 100)	Controls ( *n* = 100)	*p-value*
Maternal age (Mean ± SD) (years old)	25.6 ± 1.29	25.9 ± 1.62	0.48 a
Maternal weight (Mean ± SD) (kg)	49.39 ± 3.44	51.5 ± 2.74	0.52 a
BMI (Mean ± SD) (kg/m2)	19.64 ± 2.51	19.81 ± 2.76	0.76 a
Food habits			0.42 b
Vegetarian	56	62	
Nonvegetarian	44	38	
Socioeconomic status			0.23 b
High	2	5	
Middle	62	66	
Low	36	29	
Source of drinking water			0.76 b
Government Supply	64	58	
Private source	36	42	
Place of Residence			0.51 b
Rural	52	59	
Urban	48	41	

Abbreviations: BMI, body mass index; SD, standard deviation.

a Unpaired *t* -test.

b Chi-square test.

The *PON1* gene acts as important guardian against cellular damage from toxic agents, such as organophosphates and oxidized lipids in the plasma low-density lipoproteins.

### PON1L55M Polymorphism and RPL

The genotype frequencies of PON1 L55M were conformed to the Hardy-Weinberg equilibrium both in cases and controls. For the PON1 L55M polymorphism, the genotype frequencies of homozygous (LL), heterozygous (LM), and homozygous mutated (MM) were 19, 21, and 60% in patients with RPL, respectively; and 35, 23, and 42% in controls, respectively. The mutated allele (M) frequency was found in 70.5% in RPL patients and in 53.5% in controls. Regarding the risk of development of RPL, the LL wild type genotype and L wild type allele were taken as references. The analysis showed that the M allele was signiﬁcantly associated with an increased risk of RPL (adjusted odds ratio [OR _adj_ ] = 2.07, 95% confidence interval [CI], *p* < 0.001). The homozygous mutant genotype (MM) signiﬁcantly increased the risk of RPL (OR _adj_ = 2.07; 95%CI,; *p* = 0.011). No signiﬁcant association was observed between (LM) genotype and RPL risk (OR _adj_ = 0.88; 95%CI; *p* = 0.729). The distributions of the genotype and allele frequencies for PON1 L55M polymorphism in patients and controls are represented in Table 2 .

**Table 2:** Distribution of PON1 L55M allele and genotype frequencies in RPL patients ( *n* = 100) and control subjects ( *n* = 100)

PON1 L55M polymorphism	Cases, *n* (%)	Control subjects, *n* (%)	Adjusted OR (95%CI)	*p-value* *
Genotypes				
LL	19 (19)	35 (35)	1.0 (ref)	−
LM/MM	81 (81)	65 (65)	2.29 (1.20–4.38)	0.011
LM	21 (21)	23 (23)	0.88 (0.45–1.73)	0.729
MM	60 (60)	42 (42)	2.07 (1.17–3.64)	0.011
Alleles				
L	59 (29.5)	93 (46.5)	1.0 (ref)	−
M	141 (70.5)	107 (53.5)	2.07 (1.37–3.13)	<0.001

Abbreviations: CI, conﬁdence interval; OR, odds ratio.

* Calculated by chi-squared test; Adjusted OR odds ratio was adjusted to the age.

### PON1 Q192R Polymorphism and RPL

For the PON1 Q192R polymorphism, the genotype frequencies of homozygous (QQ), heterozygous (QR), and homozygous mutated (RR) were 51, 41, and 8% in RPL patients, respectively; and 46, 42, and 12% in controls, respectively. The R mutated allele frequency was found in 28.5% in RPL patients and in 33% in controls. Regarding the risk of development of RPL, the QQ wild type genotype and Q wild type allele were taken as references. The analysis showed that the women who were QR heterozygotes (OR _adj_ = 0.96; 95%CI; *p* = 0.887) or RR homozygotes (OR _adj_ = 0.64; 95%CI; *p* = 0.480), and R allele did not show any risk for RPL (OR _adj_ = 0.81; 95%CI; *p* = 0.329). The distributions of the genotype and allele frequencies for the PON1 L55M polymorphism in patient and controls are represented in Table 3 .

**Table 3:** Distribution of PON1 Q192R allele and genotype frequencies in RPL patients ( *n* = 100) and control subjects ( *n* = 100)

PON1 Q192R polymorphism	Cases, *n* (%)	Control subjects, *n* (%)	Adjusted OR (95%CI)	*p-value* *
Genotypes				
QQ	51 (51)	46 (46)	1.0 (ref)	−
QR/RR	49 (49)	54 (54)	0.82 (0.47–1.43)	0.480
QR	41 (41)	42 (42)	0.96 (0.55–1.68)	0.887
RR	8 (8)	12 (12)	0.64 (0.25–1.63)	0.480
Alleles		66		
Q	143 (71.5)	134 (67)	1.0(ref)	−
R	57 (28.5)	67 33)	0.81 (0.53–1.24)	0.329

Abbreviation: CI, confidence interval; OR, odds ratio.

* Calculated by chi-squared test; Adjusted OR odds ratio was adjusted to the age.

## Discussion

The key finding of our analysis is the association between *PON1* genotypes and RPL. To our information, this is the first study evaluating the outcome of polymorphisms that are involved in the genetic variability of the PON1 enzyme on RPL in north India. Toy et al. 17 approximated the relationship between the PON1enzyme and early loss of pregnancy (before the 12 ^th^ week of gestation) in pregnant women whose exposure to pesticides was unknown and found out that basal and salt-stimulated paraxonase activity were significantly lower in women who had suffered an early pregnancy failure when compared with women who continued their pregnancy beyond the 12 ^th^ week. Blanco-Muñoz et al. 18 established that the probability of miscarriage with the PON1 _192_ RR genotype was 2.2-fold higher than with the PON1 _192_ QR/ PON1 _192_ QQ genotypes. The possibility was close to 4-fold higher with the PON1 _55_ MM/ PON1 _55_ LM genotypes than with the PON1 _55_ LL genotype.

Certain authors have documented the role of *PON1* polymorphisms on other reproductive consequences, mainly gestational age, and anthropometric measures. Chen et al. 9 reported that the probability of premature delivery increases with PON1 RR genotype in newborns while studying the role of genetic polymorphism on pregnancy outcomes.

Lawlor et al., 19 in a retrospective analysis, observed that the incidence of women who complained of at least 1 spontaneous preterm delivery increased with every R allele at position 192. In Norway, Ryckman et al. 20 reported that the risk of premature birth in infants is 32% higher with the PON1–108 genotypes (OR = 1.32; 95%CI: 1.13–1.53) than in infants with PON1 _108_ CC/PON1 _108_ CT genotypes. However, no *PON1* maternal genotype was related with this.

The *PON1* gene exerts its role in lipid metabolism too, besides playing a major role in the detoxiﬁcation of organophosphorus compounds; the R isoform of the PON1enzyme shows decreased capability to hydrolyze oxidated lipids. 21 22 23 On the other hand, another study established that the level of oxidative stress increases with the QQ192 genotype. 24 Chen et al., 9 and Lawlor et al., 19 allocated the role of the RR genotype on preterm birth to damage in placental circulation coupled with lipid peroxidation. Similarly, a few studies have discovered that the presence of pesticides boosts oxidative stress and encourage the creation of oxidated lipids. 25 26 27 Accordingly, it is feasible that the RR genotype might amplify the possibility of low birthweight, mediated by placental hypoperfusion. All these studies cannot be completely compared with each other as a few focused on the genotype of the mother, while a few focused on the genotype of the infant, and others on the genotypes of both. A few evaluated the activity of the paroxonase enzyme and a few did not pay attention to it. In a few cases, the information on the exposure of the mother (either occupational or via biomarkers) was accessible, but a few studies were deficient of this variable. Nevertheless, all studies in general indicated the presence of *PON1* gene polymorphism upon adverse reproductive events and imply the existence of an interaction between miscarriage and pesticide exposure.

In contrast with a few authors who established an association between agricultural work and miscarriage, 28 29 30 we did not find an independent effect of exposure to pesticides on the risk of RPL. However, we did find an independent effect of maternal *PON1* on the risk of RPL.

Despite its limitations, the present study is one of the first in North India to evaluate the effect of *PON1* genetic polymorphism on RPL and to provide additional evidence to the increasing information regarding certain *PON1* genotypes that may affect the development of the fetus. More research is required to confirm these findings and overcome the limitations of the present study.

## Conclusion

The reduced variability in exposure of women to pesticides might explain the absence of association observed between the exposure and RPL. One limitation of our study is that we have no information available on the PON1 enzyme. This is important because even in individuals with the same genotype, this kind of activity may vary up to 13 times. 10 A large sample size might have made it possible to reach statistical significance between the genotypes and risk of RPL, as well as to show interactions between the genes and the environment.
